# Case Report: Open reduction and fixation of Pipkin type IV femoral head fracture through minimally invasive direct anterior approach

**DOI:** 10.3389/fsurg.2025.1656014

**Published:** 2026-01-05

**Authors:** Andrzej Zalewski, Mateusz Pastuszak, Jongmin Chai, Robert Jopowicz, Mateusz Kawka, Paweł Skowronek

**Affiliations:** 1 Department of Orthopedic Surgery and Rehabilitation, Bródnowski Hospital of Mazovian Region, Warsaw, Poland; 2 Department of Miniinvasive Orthopedics and Rehabilitation, Medical University of Warsaw, Warsaw, Poland

**Keywords:** femoral head fracture, MIS DAA, hip fracture, Pipkin fracture, ORIF (open reduction and internal fixation)

## Abstract

The authors present the case of a 60-year-old patient with a Pipkin type IV femoral head fracture treated surgically with open reduction and internal fixation (ORIF) using a minimally invasive direct anterior approach (MIS DAA). The patient sustained the injury in a motorcycle accident, and imaging revealed a displaced fracture of the femoral head with involvement of the posterior acetabular wall. A decision was made for surgical treatment using MIS DAA to minimize the risk of complications. The procedure was performed through an anterior approach. Intraoperatively identified free bone fragments from the posterior acetabulum wall were removed due to a high degree of comminution, and the fracture was stabilized with Herbert screws. Postoperatively, antithrombotic prophylaxis and rehabilitation were implemented. In the postoperative period, a complication in the form of deep vein thrombosis was observed, which was effectively managed. Twelve months after the operation, the patient returned to physical activity, experienced no pain, and the range of motion in the hip joint improved and consisted for 3 years of follow-up. Functional assessment using the FJS-12 scale showed gradual improvement. The use of MIS DAA in the treatment of Pipkin type IV femoral head fracture allowed for a good clinical result and avoidance of complications such as avascular necrosis of the femoral head and heterotopic ossification. This technique may be an effective alternative to traditional methods of treating this type of fracture.

## 1 Introduction

Fractures of the femoral head are infrequent high-energy injuries, in the majority caused by vehicle accidents and associated with a significant risk of long-term complications (1). The severity of the initial trauma, often characterized by femoral head destruction with acetabular involvement, fracture comminution, and associated hip dislocation, predisposes patients to adverse sequelae such as posttraumatic osteoarthritis, heterotopic ossification, and sciatic nerve injury (2). Moreover, due to the anatomical structure of the femoral head blood supply, derived mainly from medial and lateral femoral circumflex arteries, its vulnerability to iatrogenic or traumatic injury creates the risk of avascular femoral head necrosis (3, 4). According to the commonly used Pipkin classification system, femoral head fractures can be divided into four types, with type IV recognized for the involvement of the acetabular rim and associated with poorer prognosis. Due to the infrequency of femoral head fractures, available clinical studies are limited by insufficient validation, lowering the predictability of treatment outcomes (5). Among four sub-groups, Pipkin type 4 fractures represent approximately 27% of all cases (6). Poor clinical outcomes of Pipkin IV treatment have been reported because of high incidence rates of femoral head necrosis, suggesting consideration of total hip arthroplasty (THA) as a preferred surgical modality (5). On the other hand, the relatively young age of typical patients challenges this treatment option as the implant would not last the predicted lifespan, causing the need for revision in the future (7). An alternative treatment modality, open reduction and internal fixation (ORIF), is also challenging due to possible technical difficulties and the relatively high risk of jeopardized blood supply to the femoral head and neck during the accident or surgical intervention, leading to diminished outcomes (8).

Direct anterior approach (DAA) is often performed to treat Pipkin IV fracture because of the low risk of sciatic nerve injury and recurrent hip dislocation (9–12). However, due to the limited access to the acetabular posterior wall and required traction, possible injury to the medial femoral artery can occur, leading to avascular necrosis (11). Regarding the low frequency of Pipkin type 4 femoral head fractures, there is still a lack of data covering the vascular complications associated with the specific treatment modality (5). Therefore, the presented case aims to demonstrate the successful therapeutic outcome of a 60-year-old patient who recovered from a Pipkin type 4 fracture treated with ORIF via a minimally invasive direct anterior approach (MIS DAA).

## 2 Case description

**Figure 1:**
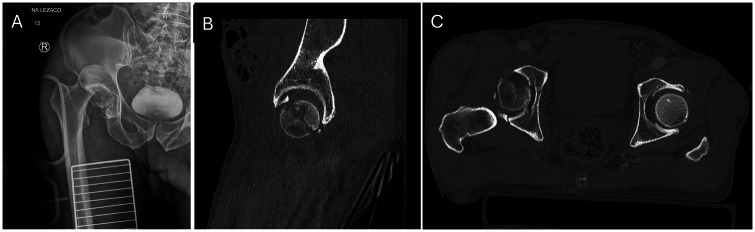
Anteroposterior preoperative radiograph of the pelvis displaying dislocation of the right hip joint after the closed reduction attempt **(A)**. Preoperative CT image with sagittal **(B)** and coronal view **(C)**, demonstrating femoral head fracture with posterior acetabular wall involvement.

A 60-year-old male with BMI 21.1 kg/m^2^ was hit in a motorcycle accident, injuring his right lower extremity. The patient was immediately transferred to the emergency department, where he reported severe pain in his right hip. The patient was hemodynamically stable and mentally oriented. Physical examination identified obvious hip deformation and peroneal nerve palsy. The patient presented with a complete foot drop, characterized by the inability to actively dorsiflex the ankle and extend the toes, while active plantar flexion was preserved. In the medical history, no previous injuries or diseases were reported. In the setting of the emergency department, radiographic examination identified posterior dislocation with femoral head fracture (Figure 1A). Immediate closed reduction was attempted and hip reduction was obtained. The patient was subsequently referred to CT examination of the pelvis which allowed identification of comminuted femoral head fracture with involvement of posterior acetabular wall, classifying as Pipkin type 4 injury (Figures 1B,C).

The decision covering the proper surgical management method was taken after prudent consideration of the patient's highly active lifestyle before the injury. Open reduction and internal fixation with Herbert cannulated screws performed via a modified minimally invasive direct anterior approach was planned instead of total hip arthroplasty. The patient was placed in the supine position. Through the modified Smith and Petersen approach (MIS DAA), an inter-muscular plane between the tensor fascia lata and the sartorius muscle was performed. During the procedure, the lateral cutaneous femoral nerve, femoral nerve and the ascending branches of the lateral femoral artery were carefully protected.

**Figure 2:**
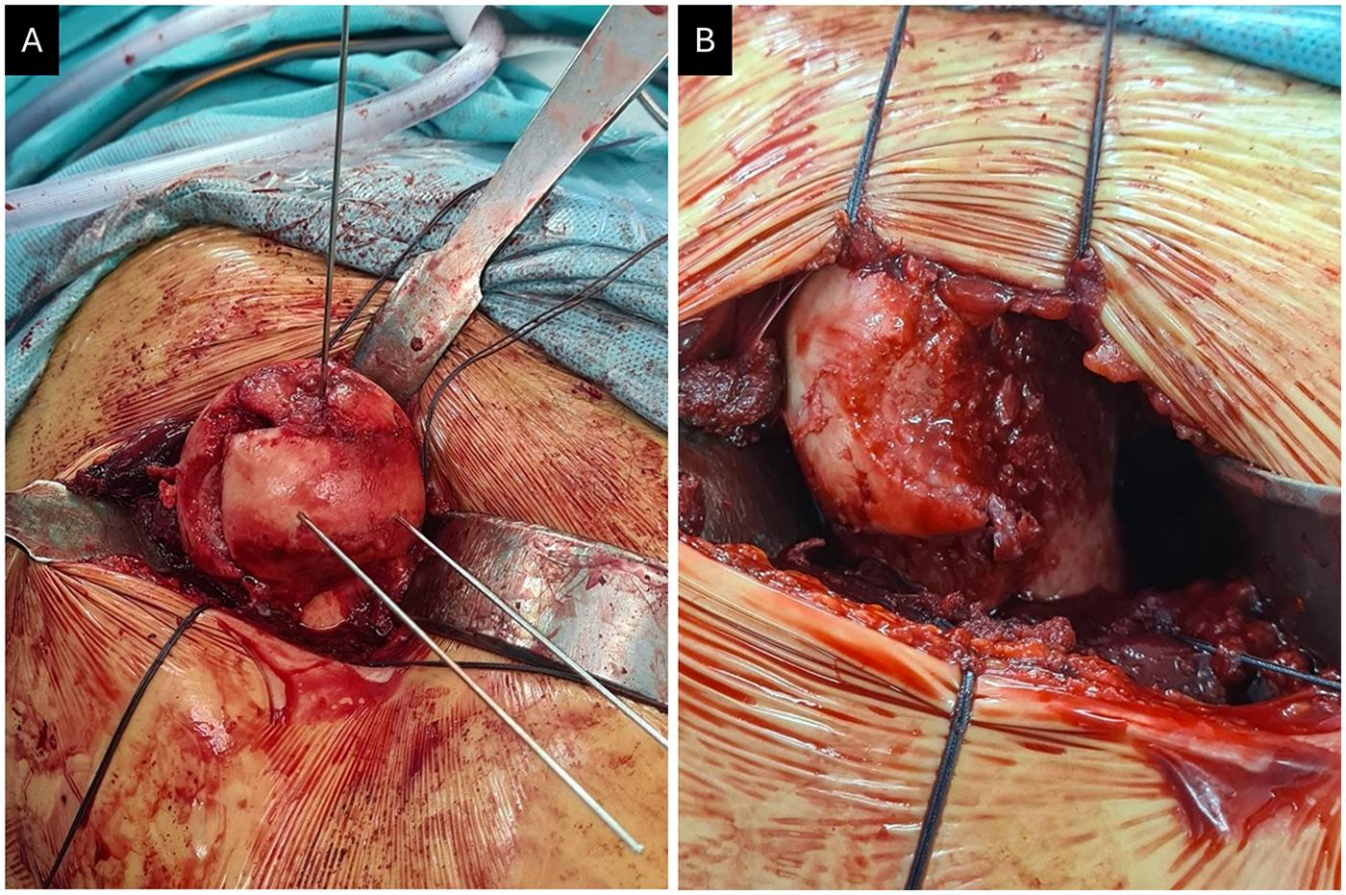
Intraoperative image of the right femoral head fracture **(A)**. Reduced fragment of the femoral head temporally fixed with K wires **(B)**.

**Figure 3:**
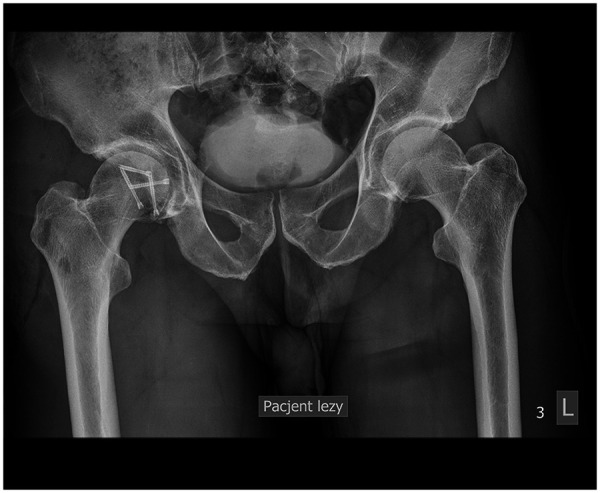
Anteroposterior radiographic image of hip joints on the first day after operation, demonstrating successful proper hip reduction with ORIF.

After dissecting the articular capsule, the hip joint was carefully subluxated under controlled traction to enhance visualization of the femoral head fracture and the posterior acetabular wall (Figure 2A). To prevent blood supply damage, the posterior part of the hip remained in a stable position, which otherwise could also deteriorate soft tissue around the posterior acetabular wall (12). Intraoperative inspection of the hip identified also ruptured ligamantum teres in the area of fracture line. Free bone fragments, identified as small, comminuted, and non-articular debris from the posterior acetabular wall, were meticulously removed from the acetabulum. This decision was made because their comminuted nature precluded stable fixation and leaving them *in situ* posed a significant risk of impingement and further articular cartilage damage. Intraoperative assessment confirmed the hip's stability after reduction and fixation of the femoral head. The fracture sites of the femoral head were temporally fixed with K wires followed by replacement with 4 Herbert screws for the internal fixation (Figure 2B and Figure 3).

Starting from the first day of the postoperative period, pain was managed with a multimodal analgesia regimen, including intravenous paracetamol and non-steroid antiinflammatory drugs, supplemented with oral opioids as needed. The Patient was also administered subcutaneous anticoagulant prophylaxis with low molecular weight heparin for 30 days. The course of immediate postoperative rehabilitation during the hospital stay included isometric quadriceps and gluteal setting exercises, active ankle pumps, and gentle active-assisted hip flexion within pain-free limits. After discharge from the hospital on postoperative day two, rehabilitation continued in the ambulatory setting. The patient was informed to avoid putting body weight on the right extremity for six weeks. After this period, a progressive low-intensity rehabilitation program was initiated at the rehabilitation center, focusing on active and active-assisted range of motion exercises, gentle stretching, and gradual weight-bearing progression under physiotherapist guidance. Although the patient received non-steroid antiinflammatory drugs as part of multimodal analgesia, which may have a prophylactic effect, no specific high-dose prophylactic regimen for heterotopic ossification was administered.

## 3 Results

**Figure 4:**
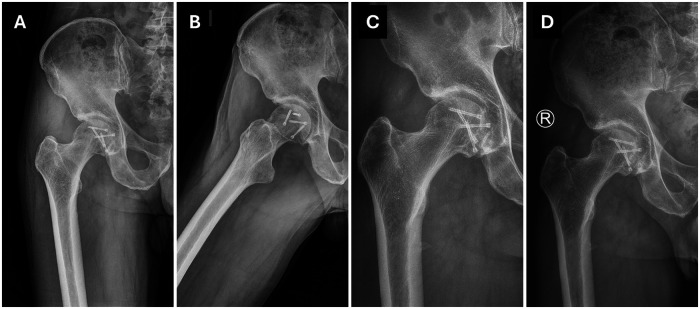
Postoperative view of the right hip showed no sign of AVN after 4 months **(A,B)**, 12 months **(C)** and 36 months **(D)** of follow-up.

One month after the surgery, during the first outpatient follow-up examination, pain in the right hip joint and foot drop from peroneal nerve injury persisted. Drop foot orthosis was implemented to support the impaired patient's gait for the prevention of stumbling and falling. At two months postoperatively, DVT was found during a routine examination on the injured side. Conservative treatment with low molecular weight heparin in therapeutic dose was introduced, which resulted in the resolution of DVT one month after introduction of the antithrombotic treatment. At three months after surgery, radiographic images demonstrated improvement in osteopenia of the femoral head with no sign of AVN (Figure 4). However, due to persistent right foot drop, the patient did not restore satisfactory gait function and was directed to the neurological rehabilitation unit.

Four months postoperatively, improved gait pattern and hip joint mobility were observed. The patient also restored the ability to extend the right toe. Detailed examination identified undisturbed sensory function at the lateral side of the right upper thigh innervated by the lateral femoral cutaneous nerve. Control radiographic analysis showed no signs of AVN.

At 12 months after the surgery, the patient started pain-free running. On physical examination, improvement in gait pattern, pain management, and restoration of ankle stability were identified. The patient was encouraged to return to an active lifestyle with further rehabilitation and remained pain-free (13). The progress in the range of motion (ROM) in the affected hip joint was determined after 36 months of outpatient follow-up (Table 1) along with standarized hip scores registered at respective time points (Table 2).

**Table 1:** Range of movement of the right hip joint after 12 months of follow-up.

Motion	Range of movement (ROM) in degrees			
	2 months	12 months	24 months	36 months
Flexion	45	90	120	120
Abduction	15	30	45	45
Adduction	15	15	30	45
External rotation	15	30	30	45
Medial rotation	15	30	30	45

**Table 2:** FJS 12 and modified Harris hip score (mHHS) of the hip joint from the patient in the timeline postoperatively.

Follow-up (postoperative months)	FJS −12 in points	mHHS in points
2 months	20	38
4 months	30	63
12 months	70	95
24 months	80	100
36 months	80	100

## 4 Discussion

Femoral head fractures are considered infrequent injuries, leading to a lack of sufficient data for the elaboration of a comprehensive classification system capable of serving as a guide for choosing optimal surgical management strategies. Among the commonly used classifications, the Pipkin classification is most frequently adopted by surgeons in clinical practice. Historically, it was developed based on observations of 24 patients with femoral head fractures. The small sample size and lack of proper validation, limit its usage for surgical decision-making guidance. Moreover, several important factors of critical importance for outcomes and management were not considered in the original Pipkin's work, i.e.,: joint congruity, femoral head fracture size and displacement or features of the associated acetabular fracture.

The patient described in this report sustained a comminuted femoral head fracture in association with a posterior acetabular wall fracture, representing a complex Pipkin type IV injury, which carries the poorest prognosis and the highest risk of complications (2, 14–16). According to the study by Shakya et al., most Type 4 Pipkin fractures were treated with open reduction and internal fixation (ORIF). This treatment was associated with a relatively high incidence of post-traumatic arthritis (43%) and heterotopic ossification (21%) (11). A key consideration in the management of Pipkin fractures, particularly in relatively young and active patients, is the choice between osteosynthesis (ORIF) and total hip arthroplasty. While THA offers immediate stability, it is associated with a finite lifespan and a higher rate of revision surgery in younger, more active individuals, which can significantly impact their long-term quality of life (11). Therefore, osteosynthesis remains a highly viable and often preferred option in this patient demographic group. The primary goal of ORIF is to preserve the native hip joint for as long as possible, thereby delaying or ideally circumventing the need for arthroplasty. Despite the inherent risks of ORIF such as avascular necrosis or post-traumatic osteoarthritis, achieving an anatomical reduction and stable fixation is paramount. Our presented case demonstrates a successful outcome with preservation of the native joint and return to a high level of function at 12 months, supporting the efficacy of meticulous osteosynthesis, particularly with techniques like MIS DAA that aim to preserve critical vascularity, as a valuable strategy to maintain joint function and delay arthroplasty in selected patients.

A matter of controversy remains regarding the optimal surgical approach for managing Pipkin fractures (7). One of the most commonly adopted surgical exposures is the posterior Kocher-Langenbeck approach; however, it often necessitates significant soft-tissue stripping to visualize the fracture, which can devitalize the head fragment and predisposes it to avascular necrosis, contributing to a relatively high rate of complications (17, 18). Other established approaches include the anterolateral (Watson-Jones) approach, which provides access between the gluteus medius and the tensor fascia lata, making it useful for accessing the anterior femoral head. For complex fractures requiring extensive visualization, the surgical hip dislocation with a trochanteric flip osteotomy, as described by Mukhopadhaya et al. (19), offers a 360-degree view of the femoral head and acetabulum with potential vascular preservation, though it adds the morbidity of the osteotomy. The conventional direct anterior Smith-Petersen approach is considered the standard for fractures requiring anterior access for the fixation of femoral head fractures, providing more muscle-sparing access to the hip, however often requires muscle elevation or partial detachment to achieve exposure. Other drawbacks and limitations have been also reported in the form of frequent occurrence of heterotopic ossification, left femoral cutaneous nerve injury or risk of vascular structures. Significant refinement of this approach, recognized as minimally invasive direct anterior modality with a smaller incision and utilization of the intermuscular and internervous plane between the sartorius and tensor fascia lata muscles, minimizes soft tissue trauma, which is critical for preserving the femoral head's blood supply, derived partially from the ascending branches of the lateral femoral circumflex artery. By carefully protecting this vasculature and reducing muscle damage, the MIS DAA theoretically lowers the risk of iatrogenic AVN and heterotopic ossification complications sometimes associated with more traumatic exposures (20).

In the presented clinical case, the direct anterior approach was performed in a minimally invasive manner, with a smaller incision length to prevent the mentioned complications. During the 36 months of follow-up, no sign of heterotopic ossification or avascular necrosis was observed. This outcome aligns with the principles of MIS DAA, which aims to minimize soft tissue disruption and muscle damage, recognized factors influencing HO risk. Factors such as patient body mass index and the extent of surgical tissue damage are also known to contribute to the risk of HO development (21). Our patient's normal BMI and the muscle-sparing nature of the MIS DAA likely contributed to this favorable outcome regarding HO. Blood vessel supply was spared in the femoral head by closely protecting the vasculature during the surgery. Another study proposed combining direct anterior and posterior approaches to secure enough surgical site visualization and reduce operation time (12). However, in our case, a single incision MIS DAA was enough to obtain sufficient access to the posterior acetabular wall and femoral head. Postoperatively, the patient maintained sensation of the lateral aspect of the right lower extremity, which could be associated with lateral femoral cutaneous nerve damage during DAA. Moreover, the patient presented with a common peroneal nerve palsy, clinically manifesting as a foot drop, which could be associated with hip dislocation due to the traction forces exerted on the sciatic nerve and its branches during the traumatic event. Implemented conservative treatment, focusing on protection with orthosis and targeted neurological rehabilitation, is a common strategy allowing for favorable outcomes (22).

## 5 Conclusion

This report illustrates a successful outcome in a challenging case of a Pipkin type IV femoral head fracture treated with open reduction and internal fixation of the femoral head only via a minimally invasive direct anterior approach. At follow-up, the patient achieved pain-free mobility and a high level of function. This technique may provide a valuable alternative for treating complex femoral head fractures, potentially decreasing the risk of complications and supporting effective rehabilitation. Meticulous assessment of the fracture location and pattern for both femoral and acetabular fractures is warranted.

## Data Availability

The original contributions presented in the study are included in the article, further inquiries can be directed to the corresponding author.
